# New products

**DOI:** 10.1155/S1463924687000300

**Published:** 1987


Journal of Automatic Chemistry/*Journal of Clinical Laboratory Automation*, Vol. 9, No. 3 (July–September 1987), pp. 135–148

### New products

### GC/FTIR

The HP 5965A is a stable, sensitive, low-cost FTIR spectrometer dedicated as a gas chromatography detector. Designed to be easy to use, it makes available capillary GC/FTIR as a routine technique in the chromatography laboratory.

Linked to the state-of-the-art HP 5890A gas chromatograph and the HP analytical workstation, the system allows chromatographers to take advantage of the most powerful combination for organic compound analysis, GC/FTIR and GC/MS. The GC/FTIR system produces wavelength chromatograms, which provide almost completely unambiguous identification of functional groups of interest.

Mass chromatograms obtained from a GC/MS system based on Hewlett-Packard's successful mass selective detector may be displayed and compared alongside GC/FTIR data, and printed out in a single report. FTIR and mass spectral library search results may be integrated to maximize the identification potential.

The unit incorporates a narrow-band mercury cadmium telluride detector which displays extreme sensitivity – it can detect five nanograms of isobutyl methacrylate at a signal-to-noise ratio of 20:1. The specially-designed, pseudo black body radiation source approaches the theoretical maximum at all wavelengths, yet has very low power consumption.

A short, straight, direct capillary interface to the GC enables peaks to elute directly to the flow cell, with a simple single fitting to minimize dead volume. The optics and flow cell path are optimized for high throughput IR transmission.

The complete GC/FTIR system is controlled from the workstation, mostly by single keystrokes. Data are displayed on the screen during the run, and integration and calculations with all standard GC methods, as well as multiple internal standards, peak ratios and multipoint calibration curves, may be used.

Spectral and chromatographic data may be displayed together, and rescale, window and zoom functions optimize interpretation and presentation. Fast library search uses databanks such as the EPA library of 3300 IR spectra, and user libraries are easy to build and search. There is a wide choice of report formats.

The GC/FTIR combination permits detection and identification of a broad range of compounds, including many that are difficult to determine by other means. In forensic science, designer drugs, with minor structural differences that cannot readily be distinguished by mass spectrometry, may be clearly identified. Isomers of hazardous pollutants in environmental samples are again readily differentiated. The ability of the system to identify trace components will be of especial relevance to the food, flavourings and fragrances industries; while pharmaceutical, industrial and environmental chemists will find particular use of the GC/IRD for fast qualitative screening of mixtures.

Optional sequencing software, with the HP 7673A autosampler, allows up to 100 samples to be analysed automatically without operator intervention.

*Enquiries to Tina Mears, Analytical Instrument Group, Hewlett-Packard Ltd, Miller House, The Ring, Bracknell, Berkshire RG12 1XN, UK. Tel.: 0344 424898.*

### Low-cost TOC water analyser

At around £10 000, the Ionics Model 555 TOC analyser is considered to be one of the lowest cost models available. It will handle a wide variety of samples, including trade effluents, seawater, drinking-water and ultra pure water.

The system has two reaction chambers; one maintained at 875°C, the other at 150°C. In the high temperature chamber, all carbon is converted to $CO_2$, which is then measured using a non-dispersive infra-red analyser. This gives a direct measurement of the total carbon content of the sample.

In the low temperature reaction chamber, only the inorganic carbon is converted to $CO_2$, thus giving the inorganic carbon content of the sample. The total organic content is simply the difference between the two results.

The Model 555 is simple to operate and maintain. Since it does not require acidification or purging of the sample, there are no pumps nor moving parts in the chemistry chain.

Sample injection is by manual syringe, sample volumes can vary according to the analysis range required. The instrument can run 0–2 ppm full scale, offering parts per billion sensitivity just as easily as 0–300 ppm for waste-water work. In both cases the typical response time would be under 2 min.

*For further details contact Ionics, Inc., 10 Statham Avenue, Lymm, Cheshire WA13 9NH, UK. Tel.: 0925 753488.*

### Dispersers

Sartorius Ltd has added two new models to the IKA Ultra-Turrax range of mixers (dispersers): the T25, a further development of the well-established T18/10 which has been used in laboratories throughout industry for more than 30 years, and the T50 which replaces the T45, a disperser that has been used successfully in pilot plants and small-scale production units, as well as in laboratories.

The T25 offers significant improvements over the T18/10: its 350 W motor is four times more powerful. This means that the operating life of the T25, under normal conditions, is even longer than that of its predecessor, despite the fact that the top speed of the T25 is 24 000 rpm as opposed to the T18/10's 20 000 rpm.

It can accommodate larger volumes – up to 2000 ml, as opposed to 500 ml with the T18/10.

It does not require a thyristor speed control in order to vary its speed. The speed control is built into the T25

and a sliding switch with four settings offers a choice of speeds from 8000 to 24 000 rpm which are extremely accurate.

The four distinct sections on the T25's sliding switch are colour-coded yellow, green, blue and red, each of which indicates a particular running speed. This means that the operator can easily repeat a particular mix at any time.

In order to cope with the larger volumes and increased top speed, two more dispersing tools have been added to the existing range. These both have 25 mm diameter rotor-stators (disperser heads) which considerably expand the range of applications. The existing range of tools fit the T25 as well as the T18/10.

The capacity of the T50 is twice that of the T45. Up to 30 l can be accommodated by the T50 at 10 000 rpm. Its motor output (drive) is 450 W compared to 390 for the T45. Again, it does not need a thyristor speed control. Like the T25, its speed control is built in.

The range of tools now available to fit the T50 is much wider than before. It includes not only heads, in a choice of diameters, for mixing or dispersing liquids, but also ones which are suitable for grinding brittle, coarse and hard materials in the wet state. The range also includes a complete unit (shaft and head) made in PTFE which is corrosion-proof and allows the T50 to be used for mixing aggressive materials.

All T50 tools have a simple push/fit method of attachment which saves the operator time when assembling the equipment.

*More information from Peter Thomson, Sartorius Ltd, 18 Avenue Road, Belmont, Surrey SM2 6JD, UK. Tel.: 01 642 8691.*

### HPLC cartridge columns

Perkin-Elmer has extended its existing range of C8, C18 and silica cartridge columns with the introduction of a fully capped C18 column. Available in 3 μ, 5 μ and 10 μ packings and a variety of lengths from 3·3 cm to 25 cm, these columns offer increased sample throughput, improved sensitivity and improved resolution, in a convenient low-cost format.

*For further information contact Perkin-Elmer Ltd, Post Office Lane, Beaconsfield, Buckinghamshire HP9 1QA, UK. Tel.: 04946 6161.*

### TLC Autospotter

A comprehensive range of TLC hardware is now available from Whatman. The range includes separating chambers, dipping chambers, UVis systems and features the Autospotter TLC sample application system.

The Autospotter is designed specifically for routine quality-control applications and allows the simultaneous application and concentration of up to 20 samples on 20 × 20 cm or 20 × 10 cm TLC plates. The sample volume can be varied between 5–50 μl. Spot diameters, after evaporation by the built-in drying system, are held between 1 mm and 5 mm.

The Autospotter is ideal for use with Whatman Linear-K, pre-channelled and conventional TLC plates.

*Details from Whatman Ltd, Springfield Mill, Maidstone, Kent ME14 2LE, UK. Tel.: 0622 61681.*

### Distilling unit for Kjeldahl and other steam distillation analyses

A typical Kjeldahl distillation takes only about four min with the Tecator 1026 Distilling unit, which perfectly matches the needs of a laboratory performing 10–50 samples per day. The 1026 Distilling unit, which is very safe and easy to operate, is the result of Tecator's long experience and know-how in manufacturing Kjeldahl equipment. It can be combined with any of the company's digestors to perform accurate and fast Kjeldahl analyses.

Dilution of the sample with water, dispensing of alkali and steam distillation for a preset time are automatically carried out by closing the safety door after placing a test-tube in the unit. All steps are microprocessor controlled and indicated with LEDs.

The steam generator heats up and is ready to use in a few minutes. It can be fed with either distilled/deionized or tap-water by the built-in pump, which also allows automatic dilution of the sample prior to the distillation.

Parameters such as alkali volume, delay and distillation time are easily preset with panel controls. This makes the 1026 distilling unit a flexible and universal apparatus, suited not only for Kjeldahl but for many general steam distillation analyses.

*For additional information contact Tecator AB, Box 70, 263 01 Höganäs, Sweden. Tel.: 46 42 42330. Or Tecator, Inc., PO Box 405, Herndon, Virginia, USA. Tel.: 703 435 3300.*

### ICP emission spectrometer

The Plasma 40 is a low-cost, bench-top sequential ICP emission spectrometer that provides, according to its manufacturer, new standards of performance and reliability. It is an integrated system controlled by an external IBM PC series (or 100% compatible) computer. Its unique free-running RF generator is built-in. There are no moving parts in the generator, so high reliability is assured and operation is almost foolproof. Even organic solvents may be sampled easily with the flip of a single switch. The plasma load coil is cooled with the same gas used for the plasma, eliminating the need for auxiliary water or gas cooling systems. Plasma 40 also includes Perkin-Elmer's corrosion-resistant demountable torch.

Plasma 40's high-resolution graphics display, extensive use of prompting, and incorporation of artificial intelligence (for functions such as automatic selection of background correction points) makes the system very easy to use. Report generation is included in the software. In addition, Plasma 40's data files are configured so that they can be incorporated easily into many commercially available data-base and spreadsheet programs to generate customized laboratory reports.

*For further information contact Perkin-Elmer Ltd, Post Office Lane, Beaconsfield, Buckinghamshire HP9 1QA, UK.*

### Centocor

Located in Leiden, The Netherlands, Centocor Europe B.V. is a biotechnology company, which develops, manufactures, and markets diagnostic and therapeutic products for the management and treatment of patients suffering from cancer, as well as cardiovascular and infectious diseases.

Centocor Europe is a fully-integrated biopharmaceutical manufacturing facility. Spanning 45 000 $ft^2$, this facility is the first of its kind to settle in the Bio-Science Park on the University of Leiden campus. Construction of Centocor Europe began in 1985, and within 13 months the manufacturing facility was ready for operation.

The production facility contains complete support systems for monoclonal antibody-based manufacturing. According to Good Manufacturing Practice (GMP), these systems include the sterilization of all materials used in production, the preparation of growth medium, the fermentation of cell lines for the secretion of monoclonal antibodies, the purification of monoclonal antibodies, and the quality control of the entire manufacturing process.

Centocor's products are based on monoclonal antibodies which are natural proteins that bind specifically to foreign substances in the body. The products consist of *in vitro* blood tests, diagnostic imaging agents, and therapeutics. The various blood tests detect certain types of breast, ovarian, and gastrointestinal cancers.

Centocor's imaging agents also aid in the diagnosis of these cancers as well as cardiovascular diseases. One of these products, Myoscint, is the world's first monoclonal antibody-based cardiac imaging agent. It identifies the location and extent of myocardial necrosis in the early hours after an acute heart attack. Knowledge of irreversibly damaged heart tissue may result in more efficient patient management. Centocor is currently developing an imaging agent which may detect the existence, location, and size of blood clots.

Centocor's therapeutic line consists of an anti-platelet antibody that may aid in the prevention of blood clots. Other therapeutic products include Panorex, which may aid in the treatment of pancreatic cancer, and Centoxin, which may aid in the treatment and prevention of toxic shock, caused by the secretion of gram negative bacterial endotoxins. Centocor's diagnostic imaging agents and therapeutic products are currently in advanced clinical trials at major research centres throughout Europe and the USA.

To develop, manufacture, and market these products, Centocor has assembled a team of skilled professionals, who are trained in medicine, biochemistry, and pharmacy. Centocor Europe B.V. currently markets its products in certain European, Middle Eastern, and Asian countries.

Centocor Europe B.V., founded in 1984 to provide healthcare products for a worldwide market, is a joint venture of Centocor, Inc. in Malvern, Pennsylvania, and the Maatschappij voor Industrieele Projecten (MIP) in The Hague, The Netherlands.

### On-line radioisotope monitoring

Beckman have introduced the 171 radioisotope detector for drug, pesticide and metabolism studies, receptor binding analysis, production purification, hybridization probe desalting and protein mapping. The fast, accurate detector, which is cold room compatible to protect labile compounds, provides quantitation of single and dual-labelled compounds as they are eluted from a liquid chromatography column. By adding a chart recorder, fraction collector or printer, total automatic operation and final answers are possible.

The 171, which can be used as part of Beckman's recently introduced 'System Gold' HPLC Personal Chromatography System, has eight independently programmable user files for minimal interaction by research investigators and features preset isotope settings for tritium, carbon-14, phosphorous-32, sulphur-35 and iodine-125, automating adjustment for any other chosen isotope by means of the 'Auto-Isoset' feature. A unique cartridge design flow cell allows quick volume changes and easy decontamination. The 'Cell-Set' facility provides self-adjusting circuitry to optimize counting conditions when using packed cells or liquid cells.

The new detector is available as either a solid system, liquid system or both. The solid scintillator – Ultra-Scint – combines maximum counting efficiency for tritium whilst remaining inert to most compounds. For the liquid flow cell, three cocktails have been formulated to maximize counting efficiency from any column effluent – 'Ready-Flow II', 'Ready-Flow III' and 'Ready-Organic'.

Standard product features include a four-line by 40-character liquid crystal display, menu-driven programming with HELP screens, automatic fraction collector control, luminescence correction, automatic background subtract, auto start and stop and user-accessed diagnostics. Options include printer, UV tracing capability, Integration and Peak-Plot program with printer, stream splitter and high efficiency mixer for the liquid system, computer output (RS232C) and 'ChromatoGraphic' software for the IBM Personal Computer.

*Details from Beckman Ltd, Progress Road, Sands Industrial Estate, High Wycombe, Buckinghamshire, UK.*

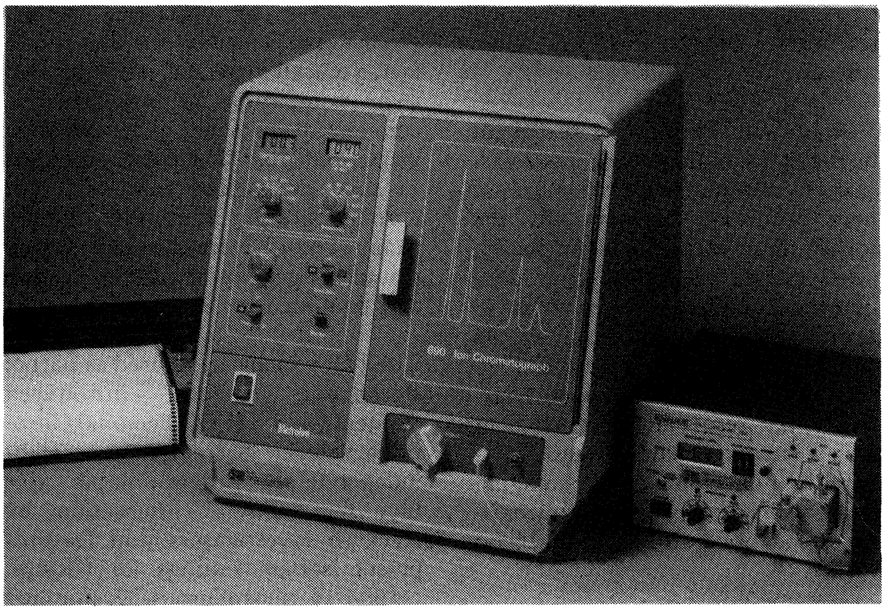

*The Metrohm 690 ion chromatography (Roth Scientific). Applications include: determination of chloride, nitrate and sulphate in rain water; determination of chloride, nitrate, phosphate and sulphate in feed and treated water; determination of chloride, nitrate and sulphate in drinking-water; determination of nitrate, phosphate, sulphate and oxalate in a vitamin enriched, mixed fruit drink (unsweetened, 10 fruits, 10 vitamins); determination of chloride, nitrate, phosphate and sulphate in a chromium (VI) plating-bath; determination of extreme ratio of calcium and magnesium in 100 : 1 ratio; determination of calcium and magnesium in tap-water; determination of lithium, sodium, ammonium and potassium in mineral water; determination of zinc, magnesium and calcium in one run; determination of anions in pine-needles.*

### Ion chromatograph

Roth Scientific, the UK distributor of Metrohm laboratory equipment, can now supply a new ion chromatograph. The Metrohm 690 Ion Chromatograph simplifies the detection of low concentration ions and allows the rapid production of high-quality chromatograms. The IC 690 enables the analyst to determine quantitatively and simultaneously an extensive range of analytes at trace concentration. The instrument has applications in a diverse range of fields, especially energy, chemicals, pharmaceuticals and environmental.

Features of this single column ion chromatograph include – the ability to use a wide range of ion-exchange columns, eluents and pH, providing the analyst with a flexible approach to system optimization. Additionally, this configuration is inherently simple with low reagent consumption and giving a cost-efficient performance.

Metrohm's 690 Ion Chromatograph conductivity detector is housed in a thermally insulated Faraday cage and mounted on a mobile vertical track to ensure optimized connections between the column and detector. The detector cell is thermostatically controlled to greater than +/- 0.01 °C producing good base-line stability.

A true auto-zero facility at the 690 IC output (as opposed to the integrator or pen recorder input) effectively allows background conductivity to be 'electronically suppressed'. The auto-zero can of course be triggered externally by, for example, an integrator or auto sampler.

The design of the 690 'wet section' allows the user easy access and maximum flexibility in the selection and maintenance of components such as the column, injection loop and detector. Sufficient space is available inside this housing for other column configurations and additional detector cells, for example, an electrochemical detector.

The layout of the front panel enables working parameters to be seen at a glance, with the dual liquid crystal displays providing information about the selected sensitivity and the conductivity of the eluent. At the rear of the instrument are two independent outputs of 1V or 10mV fsd for connection to a datalogging system such as a pen recorder or integrator.

*Details from Roth Scientific Co. Ltd, Alpha House, 9–11 Alexandra Road, Farnborough, Hampshire GU14 6BU, UK. Tel.: 0252 513131.*

### Two-wire pressure transmitter range

A low-cost range of two-wire pressure transmitters providing a 4–20 mA signal for gauge, absolute and differential pressures of air and gases, water and liquids has been introduced by the Perflow Instruments.

The new instruments (the Percept range) are claimed by the company, which specializes in flow and low pressure measurement technology, to be the most compact currently available. Designed for remote indication and for control purposes in medium-duty applications in building services, gas, water and dust extraction plant, the instruments employ high specification IC transducers to provide a wide sensitivity range combined with consistent accuracy and reliability.

The new Percept instruments are sensitive to conditions extending from 0–2.5 mbar full scale, to 0–7 bar of gauge, absolute and differential pressures.

Two versions are available: the HE model for low pressure air and non-corrosive gases which is contained in a die-cast housing sealed to IP65; and the WE model for wet/wet differential applications, which is provided with a specially designed module which incorporates equalizing and over-pressure relief valves to give protection from the effects of pressure surges or mishandling. The protective module is secured directly to the electronics enclosure.

Percept two-wire transmitters are designed for use with averaging pitot, venturi and other low-loss flow elements as well as conventional orifice plates. All are designed, manufactured and calibrated by Perflow Instruments Ltd.

*Information from Perflow Instruments Ltd, Chapmans Park, High Road, London NW10 2DY. Tel.: 01 451 4577.*

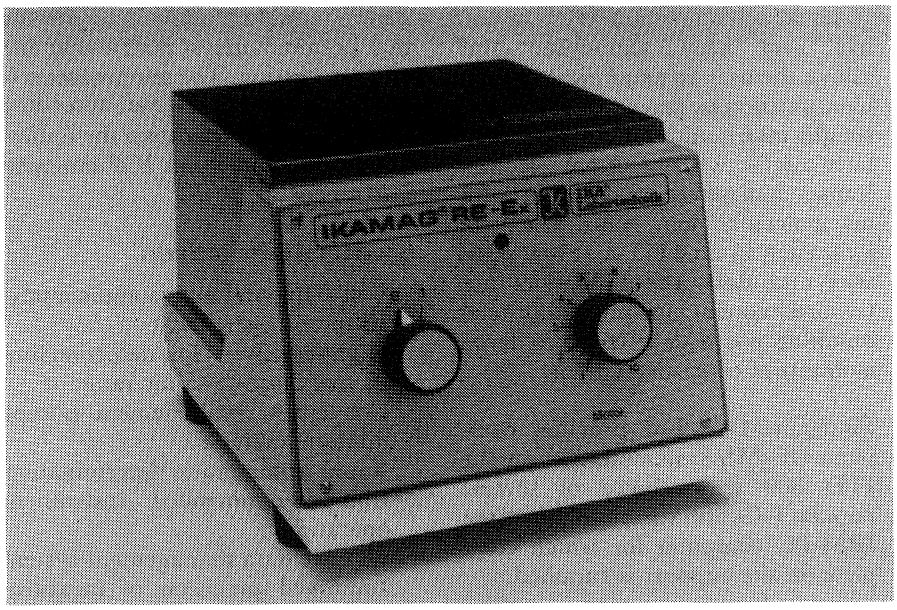

*Sartorius Ltd, of Belmont, Surrey, UK, has launched an explosion-proof version of the IKAMAG RE magnetic stirrer. Robustly constructed, it has a compact flameproof enclosure which measures 150 mm (w) × 230 mm (d) × 125 mm (h). The stirrer (RE-Ex) carries the maximum safety temperature classification, i.e. T6, and conforms to EEC standards for equipment operating in hazardous locations. The RE-Ex has a speed range of 50–700 l/min and the chosen speed is infinitely variable within this. Suitable for handling volumes of up to 5 l, it is highly stable, due to its solid construction, and can accept loads as high as 100 ks. More information from Peter Thomson, Sartorius Ltd, 18 Avenue Road, Belmont, Surrey SM2 6JD, UK.*

### LDC/Milton Roy to sell Spectronic instrument range

Following the formation by Milton Roy of its European Laboratory Group in January 1987 the company has now announced the addition of the 'Spectronic' range of spectrophotometers. Previously LDC/Milton Roy has concentrated on HPLC products, and the incorporation of the 'Spectronic' range (ex. APD/Bausch & Lomb) is an interesting and signficant broadening of the company's product base.

The 'Spectronic' instrumentation consists of a series of state-of-the-art spectrophotometers starting with the familiar Spectronic 20 and its latest version the 20D – by far and away the most widely used instrument in the world, with over 100 000 sold – through the 501 and 601 models (now available in enhanced performance versions 501C and 601C), on up to the powerful Spectronic 1201 which allows scanning up to 3000 nm per minute.

The 'Spectronic' instrumentation will be available throughout LDC/ Milton Roy's established sales network both in the UK and in continental Europe and will be backed up by the company's field service capability.

*For further information contact LDC/ Milton Roy, Stone Business Park, Stone, Staffordshire ST15 OHH, UK. Tel.: 0785 81352.*

### Ion trap detector

The new Model 800 ion trap detector (ITD) from Finnigan MAT provides lower detection levels than other mass spectrometric detectors, while scanning the full mass range throughout a GC run. Competitive detectors using quadrupole technology depend on measurement of a few selected ions for low sample levels, sacrificing specificity for sensitivity.

The specific mass spectrum which results from the Model 800's full-scan operation adds another important level of certainty to identification in GC at trace detection levels. This is especially advantageous in applications with potential legal implications. Furthermore, the ITD 800's extended dynamic range allows quantitative GC analysis over a large concentration range, using internal or external standards for highest precision and accuracy.

These achievements in full-scan sensitivity and dynamic range are attributed to new Automatic Gain Control (AGC) software that continuously adjusts the ITD's operating parameters, based on the amount of sample eluting from the GC column into the detector. The AGC algorithm measures the amount of sample present at a given moment and adjusts the ionization time in micro-seconds to produce optimum results.

Full mass scan sensitivity on the Model 800 is improved by orders of magnitude over conventional mass detectors resulting in unambiguous detection and identification at sample levels as low as 10 picograms. Linear dynamic range covers four to five decades, from low-picogram to high-nanogram levels of samples. Finnigan MAT has announced that the AGC software is being made available to all present owners of the earlier Model 700 ITD.

The ITD can replace other specific detectors or a flame ionization detector (FID) on any capillary gas chromatograph. It produces a chromatogram and mass spectrum for a GC analysis, providing both retention time and mass spectral information for quantitative and qualitative analysis.

A chemical ionization (CI) option has also been announced – this allows the user an extra dimension of identification by providing molecular weight information. This is particularly useful where normal electron impact ionization produces little or no molecular ion. Since chemical ionization in an ITD is at low pressure and flow, the ITD does not require frequent cleaning, additional pumping nor does it consume large amounts of expensive CI gas.

Finnigan-MAT Ltd provides complete GC-MS systems based on the ITD 800 with choice of several popular GCs and various levels of the IBM-PC computer for which complete on-site support is supplied.

The Model 800 ITD is priced at £28 000 in the UK. An IBM PC/XT or PC/AT computer is used for instrument control, data acquisition and report generation. With the PC/XT the ITD is priced at £32 250, with the PC/ATX it is £33 500. Total system packages, including a capillary gas chromatograph, Model 800 ITD and computer, are priced from £36 000 to £50 000 depending on specification.

*More information from Finnigan MAT Ltd, Paradise, Hemel Hempstead, Hertfordshire HP2 4TG. UK. Tel.: 0442 233555.*

### ICP-MS System

The Elan inductively coupled plasma-mass spectrometer is the first product of a joint venture between Perkin-Elmer and the Sciex Division of MDS Health Group Limited, of Thornhill, Ontario, Canada. Manufactured by Sciex, the instrument will be marketed, serviced and technically supported by Perkin-Elmer. It is designed for rapid multielement determination of elemental species at low concentrations and is complementary to both ICP emission spectroscopy and graphite furnace AA.

The configuration of the Elan is similar to that of an ICP emission spectrometer: consisting of a plasma source, a spectrometer, a detection system, and a computer/controller that permits analyst interaction and data processing. The spectrometer of the Elan disperses ions according to their mass, rather than the optical dispersion used in an ICP emission instrument.

Special features include:

- Full elemental and isotopic analysis in one instrument
- High sensitivity, low detection limits and large dynamic range
- Convenient multielement isotope dilution analysis
- Rapid isotope ratio determination
- Computer-controlled instrument operation
- Flexible data management system
- Improved precision of measurement with pulse counting
- Negative ion capability for determination of F, Cl and S.

*For further information contact Perkin-Elmer Ltd, Post Office Lane, Beaconsfield, Buckinghamshire HP9 1QA, UK.*

### Bioassay

Labsystems have developed an instrument that can perform bioassays for any compound that affects the growth characteristics of micro-organisms.

Bioassays are traditionally performed as end-point assays. The compound to be measured is normally a growth-limiting factor for the test micro-organism. The effect of the compound on the final growth level of a culture is interpolated using a standard curve.

Bioscreen measures kinetically a number of parameters relating to microbial growth. The characteristic that best relates to the concentration of the substance is utilized. Since the data is obtained throughout the growth curve it is possible to reduce the length of time taken for a bioassay while increasing its accuracy and precision. A kinetic assay will also provide information not made apparent by an end-point test about the effects of the test substance on the rate of growth.

The Bioscreen consists of an aseptic dispenser/diluter, incubator/mixer, photometric system and data processor. All steps of the bioassay are automated from sample addition through to data analysis and report production. The software is sophisticated and has been designed by Labsystems to be used in a straightforward manner.

*Further information from Labsystems (UK) Ltd, 12 Redford Way, Uxbridge, Middlesex UB8 1SZ, UK. Tel.: 0895 38421.*

### Elemental analysis

Philips Analytical announced two major innovations in the atomic absorption technique in Cambridge, UK in May 1987. The company already has a good share of the AA market with the SP9 and PU9000 series of instruments – changing market demand led the group towards the development of the new PU9200 and 9400 series.

### *The PU9200 series*

The series was designed with total flexibility in mind. It offers a wide choice of cost-competitive, factory-configured instruments backed up by an extensive new accessory range so that the analyst can build a laboratory-tailored system in one integrated package – and expand easily when requirements change or budgets allow.

With fully automatic parameter set-up as standard, instruments in the PU9200 Series offer the user variety of system configurations: single-beam or Stockdale double-beam optics, automatic or full gas control systems, single (two element) or quad (eight element) data-coded lamp turrets, routine or extended operating software and manual or autosampling facilities; a dedicated furnace instrument is also available.

A full range of new and advanced accessories means that each basic instrument can be upgraded to suit changing requirements. Options include a new furnace, furnace autosampler, furnace autoprobe, integral flame autosampler, continuous-flow vapour system, slotted tube atom trap and disk storage.

The PU9200 Series furnace system can be interchanged rapidly with the burner system without disconnecting gas lines. Providing an all-graphite, inert cuvette environment, the furnace possesses the most comprehensive interference control package available – including the unique Furnace Autoprobe which minimizes vapour phase interferences. Furnace autosampling – together with automatic sample preparation – is available with the PU9380 autosampler, which allows lengthy, unattended analyses to be performed with ease.

Built into the PU9200, the optional PU9302 flame autosampler may be used with routine or extended operating software. Features available include all standards cocktailing modes, automatic aliquot sampling, recalibration, sample vial sensing and programmable wash facilities.

Automatically self-cleaning, the vapour system brings the benefits of continuous, rather than transient, signals during the measurement of hydride-forming elements and mercury. The unique slotted tube atom trap (STAT) provides enhanced flame sensitivity, bridging the flame/furnace gap for elements such as As, Se, Pb, Cu, Zn and Cd.

Every instrument in the PU9200 range offers a high level of performance. The flame system – which provides the finest sensitivity and precision of any instrument available – employs an advanced fluorocarbon spray chamber. Improved laminar flow burners give high solids handling capacity and excellent flame stability, and the gas system is controlled via programmable array-state logic, with comprehensive interlocks including the prevention of use of incorrect flow rates or ratios.

Single or multi-element data-coded hollow cathode lamps and minimum component single-beam or Stockdale double-beam optics provide a measurement system of the highest pedigree. High-energy, beam-profile matched background correction is standard, and each instrument has a specified performance up to a full 2A of background with an error of less than 2%.

The user interface of the PU9200 employs a QWERTY keyboard, and all operation is via simple menu-driven options, with help information available at the touch of a dedicated function key. Results are presented on a high-resolution 12 in VDU, and the addition of a printer provides the facility for hard copy reporting.

Routine software operates the flame and autosampler, while optional extended software covers all working modes – flame, furnace and vapour. Both software sets have high-resolution graphics facilities and only present relevant choices and parameters for a particular mode.

Optional extension of a large capacity dual 640 kB on-board disk system is rapid and straightforward, giving facilities for 16 element analyses, data manipulation, and results, graphics and methods storage.

*PU9485*

Philips describe the PU9485 as a 'massive leap forward' in intelligent, automatic multi-element atomic absorption spectrometry. The 9485 has the capacity to determine up to 16 elements sequentially and offers state-of-the-art data manipulation and storage facilities in one integrated unit.

The PU9485 automatically adjusts flame conditions and, dependent on the signal level from the sample matrix, will select the optimum combination of analytical parameters including lamp current, read time, bandpass and flame conditions. Should the analyte be too concentrated, a suitable alternative wavelength will be selected. As a result, standard conditions which may not always apply from day to day may be individually optimized for each analysis without operator intervention.

The PU9485 in the world's first AA spectrometer with an onboard, high-resolution colour VDU. Fast and easy to read, it gives immediate differentiation between keyable and status parameters. Status messages have instant meaning, and considerable visual resolution is added to multiple graphics plots.

Methods, graphics and results are stored on twin, high-density 640 kB disks – a design that allows complete analysis parameter sets to be stored, assembled into working multi-element tasks and run at a keystroke. Furthermore, the disks provide full-page user guides for the important working VDU pages – for example Stored Task Sequence Definition, Optical, and Flame Set-up – and should the operator need to record written notes on an analysis, memo pages may be stored with the appropriate method.

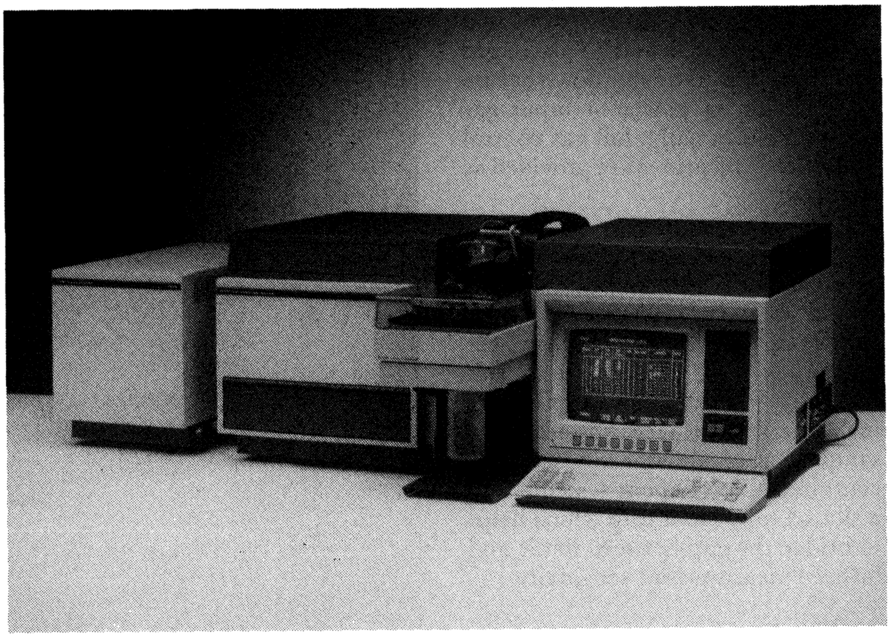

*The PU9200 Series AA Spectrometer. Furnace autosampling, together with automatic sample preparation, is available with the PU9380 autosampler.*

Software for all working modes is standard, reducing the changeover time from one analysis technique to another to a minimum. The PU9485 will even suggest how the autosampler should be loaded for an analysis, and diagnose the fault should a problem occur.

All results are presented in real-time, in either tabular or graphical form, on the integral colour VDU. Full status is always presented, and graphical features may be recalled within the instrument software by overlaying any three required signal displays for comparison – a useful facility in method development or results auditing. Addition of a printer provides not only the normal results and parameter print-out, but at the touch of a special function key, hard copy of any page within the system.

Other features include 'Stockdale' double-beam optics and pre-aligned data-coded hollow cathode lamps. A high-energy, beam-profile matched continuum source background correction system is modulated at an optimum 150 Hz with little more than 2 ms between measurements, offering correction up to 2A of background with an error of less than 2%.

The flame system employs an advanced fluorocarbon spray chamber, while improved laminar flow burners give high solids handling capacity and good flame stability. A binary switching flow control system is used to produce precise and accurate gas flow, and automatic shutdown occurs in the event of power or input gas failure. Additionally, full gas control system safety features are provided as standard.

A full range of new and advanced accessories – all fully compatible with Philips new PU9200 Series of AA spectrometers – means that the PU9485 can be configured to suit changing requirements. Options include a new furnace, furnace autosampler, integral flame autosampler, continuous-flow vapour system – and a slotted tube atom trap which helps to bridge the gap between flame and furnace measurement sensitivity.

Providing an all-graphite, inert cuvette environment, the PU9485 furnace system posesses the most comprehensive interference control package available – including a Furnace Autoprobe which minimizes vapour phase interferences.

Furnace autosampling – together with automatic sample and standards preparation – is available with the PU9380 autosampler, which allows lengthy, unattended, multi-element analyses to be performed with ease and at the ultimate levels of sensitivity.

Built into the PU9485, the optional PU9304 flame autosampler is compact and space-saving. Direct control by the central 10 MHz 16/32 bit microprocessor makes even complex tasks remarkably easy to perform. Features include all standards cocktailing modes, automatic aliquot sampling, automatic reblanking, rescaling and recalibrating, automatic sample vial sensing and programmable wash facilities. All the operator needs to do is slide in the autosampler carousel, recall the analysis task from disk and press the start key for the most complex of multi-element analyses with optimum results.

*AA literature*

A comprehensive range of AA support literature has been published by Philips Analytical to coincide PU9200 and PU9400 launches. Five methods development papers describe protocols which will be of value to analysts in a number of applications areas. Topics covered include the determination of trace elements in copper, alloying agents in steel, metals in lubricating oils, and major/minor electrolytes in serum.

Covering sampling, sample/standard preparation and instrumental parameters, the papers illustrate the felxibility and capability of the new instruments – including high solids handling and multi-element tasking.

Philips has also published five papers which cover aspects and design considerations of modern AA instrumentation. Areas featured include graphite furnace technology, the furnace autosampler, flame atomization systems, graphite probes and the slotted tube atom trap.

*Analytical software*

A software package for production and quality-control applications, which provides cost-effective sample data management for multi-instrument laboratories, has also been announced by Philips.

Called LAB60, it runs on DEC's PDP11 family of computers and is primarily intended for use with Philips X-ray and optical emission instrumentation. However, it can also be used with any equipment capable of delivering a suitable ASCII output.

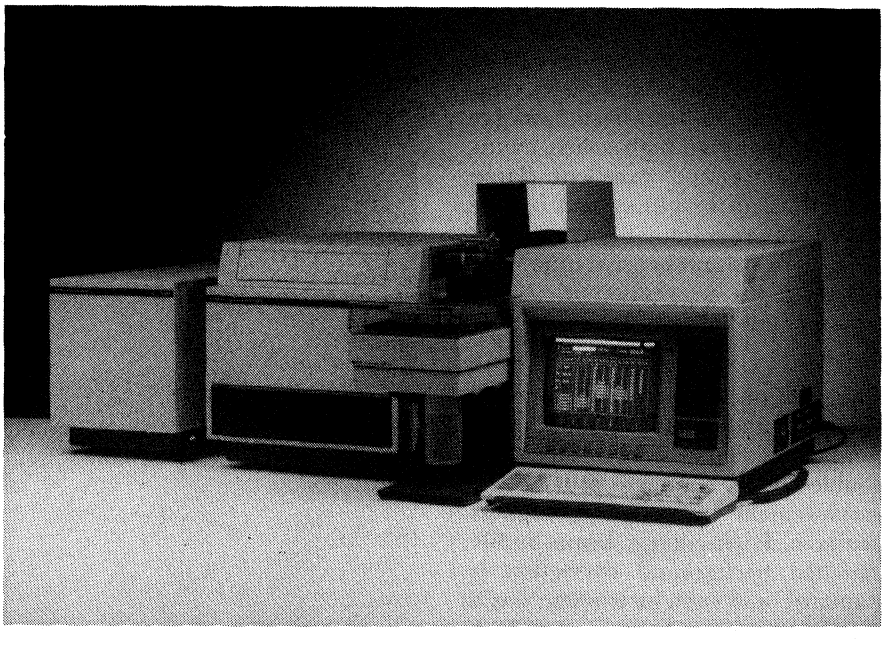

*The PU9400 Series spectrometer, featuring Philips Analytical's PU9380 furnace autosampler, which allows lengthy, unattended analyses to be performed with ease.*

Results from various on-line and off-line sources can be merged to give a complete and prompt analytical report, available via video terminals or hard-copy printers in the laboratory or at remote locations.

LAB60 is offered alongside the Philips Automated Laboratory Management (PALM) software package which provides additional facilities such as extended data-base manipulation and a more flexible sample identification system.

The layered structure of LAB60 software ensures that urgent operations are given priority status. An active data-base retains full information on all samples of current interest, ensuring high speed access to results and immediate indication of sample backlogs. Less urgent data for archiving is transferred to a second level – the passive data-base.

Operator communication is via easily understood menus and dialogues, using the same style and language as that of the PALM software package. Help messages can be called up should problems arise, and extensive error indications and fault warnings provide further safeguards against mistakes.

Optional modules for reporting and statistics, quality search and charge correction extend LAB60's data processing capabilities still further, enabling the package to meet the needs of a wide range of primary materials producers and manufacturing industries.

*Brochures about Philips Analytical's new products from Pye Unicam, York Street, Cambridge CB1 2PY, UK. Tel.: 0223 358866.*

### 'Troubleshooting HPLC systems'

*Troubleshooting HPLC Systems* is the title of a video course written and presented by John W. Dolan and Lloyd R. Snyder and distributed outside North America by Elsevier Science Publishers, Amsterdam. The course consists of three 55-minute videotapes aimed at improving the HPLC problem solving skills of practising chromatographers. The tapes cover: Principles of troubleshooting; Fittings, reservoirs, pumps, and injectors; and Columns, detectors, and preventive maintenance.

The course is ideally suited for self-tuition, class use and in-house training courses, making expensive external courses unnecessary. The number of service calls will also be reduced.

A 100-page *User's Manual* contains copies of 150 visuals used in the video, so note-taking is easy. There is also a short *Instructor's Guide*, which contains tips on using the course in a classroom.

The complete course includes one of each of the VHS tapes, a *User's Manual* and an *Instructor's Guide*. The cost is 3000 Dutch guilders postfree, but does not include possible customs duties and taxes. Multiple copies of the *Manual* and *Guide* may be ordered. The publisher is offering a Demonstration Tape at 50 Dutch guilders, prepaid. This 25-minute tape contains samples from all three tapes.

*Details from Elsevier Science Publishers, PO Box 330, 1000 AH Amsterdam, The Netherlands.*

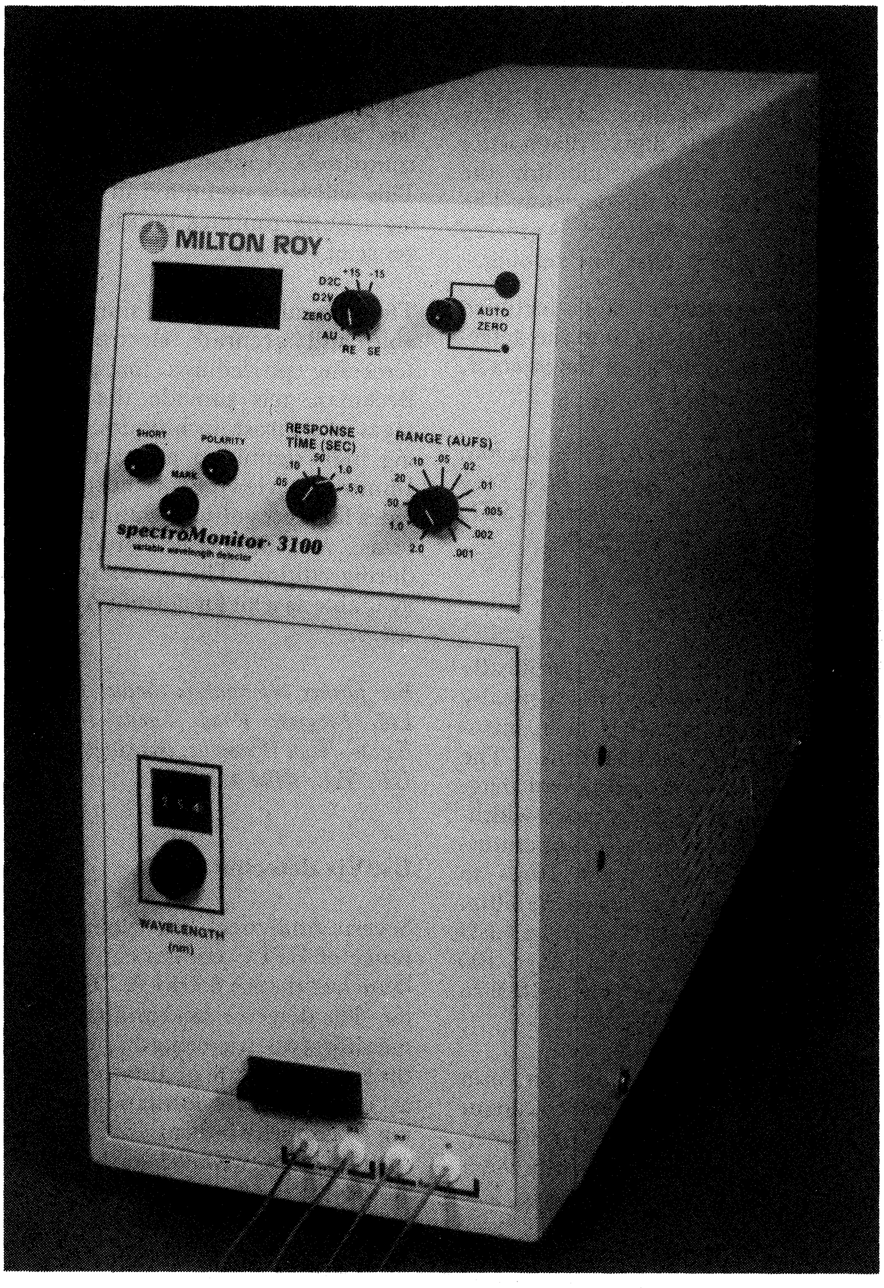

*SpectroMonitor – a low-cost variable wavelength detector for HPLC.*

### HPLC

SpectroMonitor 3100 (see p. 143) is a variable wavelength detector for HPLC announced by LDC Milton Roy on 1 June 1987. The spectro Monitor 3100 features linear operation from 2·0 AUFS to 0·001 AUFS. Typical noise performance is $2 \times 10^{-5}$ AU peak-to-peak at 254 nm, providing excellent detection capability in the 0·001 AUFS range. Absorbance measurements can be made throughout the entire UV-visible range (190–700 nm). A 10 mm pathlength maxN Series high-efficiency, high-pressure (1000 psi) fluid cell is standard in the spectroMonitor 3100 detector. Optional fluid cells for preparative, semi-preparative, and microbore HPLC are available.

The spectroMonitor 3100 also features complete digital diagnostics allowing the user to monitor the operating status of the detector. The one-step, push-button Autozero feature simplifies base-line zeroing.

An injection valve bracket is included for mounting directly onto the detector for close coupling of the injector, column and cell.

*More information from LDC/Milton Roy, Milton Roy House, Diamond Way, Stone Business Park, Stone, Staffordshire ST15 0HH, UK. Tel.: 0785 813542*

### Micropump drive

A new drive, developed specifically for the Micropump magnetically driven gear pumps, has been introduced by Watson-Marlow. The 5003U drive has a permanent magnet d.c. motor driven by pulse-width-modulated circuitry, and is operator-driven through feedback loops to ensure accuracy and speed stability. Motor speed is displayed on an LED and controlled over the range 100 r.p.m. to 5000 r.p.m. via a 10-turn potentiometer.

The 5003U drive can be user programmed to accept any analogue process signal up to 30 V or 30 mA, or can be controlled manually via a front-panel flow-control switch.

Accepting a range of micro-pumps the 5003U gives flow rates of up to 4600 ml/min, system pressures up to 20 bar and differential pressures up to 8 bar.

Micropumps share many of the advantages of peristaltic pumps – self-priming, dry running, non-contaminating and a positive displacement action. They become the preferred pump type when pressures higher than a continuous 1·5 bar are required at flow rates up to 4600 ml/min, or when fluids, such as solvents, which are not compatible with peristaltic pump tubing, are to be handled.

*For further information contact Smith and Nephew Watson-Marlow, Falmouth, Cornwall TR11 4RU, UK.*

### Gel view box

Beckman has added a new gel view box as a standard accessory to the company's Appraise densitometers. This will be a useful aid in performing editing routines by viewing the gel scan.

The Appraise provides simple, reliable and accurate electrophoresis screening procedures for proteins. Beckman gels provide the advantages of agarose techniques, including high resolution and sensitivity. Immediate viewing of the gel provides fast recognition of unusual patterns, and increased productivity during editing. The gel view box is available as a kit for current Appraise users.

*For further information contact Beckman Ltd, Progress Road, Sands Industrial Estate, High Wycombe, Buckinghamshire, UK. Tel.: 0494 41181.*

### Uv/Vis detector

Severn Analytical has expanded its range of HPLC equipment with the launch of the SA 6504 UV/Vis detector. The detector can simultaneously monitor two wavelengths, enabling the user to acquire qualitative as well as quantitative information including peak purity indication. Chromatograms at both wavelengths can be stored by the detector for replay at variable speed and sensitivity. In addition, the detector can also plot the ratioed chromatogram. Peak detection software is also a standard feature of the SA 6504, permitting automatic control of fraction collectors.

*Details from Severn Analytical, Unit 2B, St. Francis' Way, Shefford Industrial Park, Shefford, Bedfordshire SG17 5DZ, UK. Tel.: 0462 815892.*

### Tablet dissolution systems

Philips Analytical has developed a series of tablet dissolution monitoring systems based on ultraviolet/visible spectrophotometry. Central to these systems, which have been produced in collaboration with the pharmaceutical industry and meet all British and US pharmacopoeia regulations, is a purpose-built, multitasking software package. Whether a simple dissolution profile in real time or quantitation of the curve shape is required, the software will provide fast, easy-to-use methods and present analytical answers in the report format of the operator's choice.

Trouble-free tablet dissolution is also dependent on repeatedly high-grade results, and Philips Analytical's new PU8620 series of UV/VIS/NIR spectrophotometers are being promoted in market for tablet dissolution.

All systems rely on the precision built eight-cell programmer, which accommodates six samples, together with standard and blank if required. The system uses continuous flow operation, thus providing fast continuous display of data. Separate channels ensure no cross-contamination between vessels.

Programming is extremely flexible, and measurements can be made at fixed or variable time intervals. For example, in a typical hour's run one could either take six readings at equal 10-minute steps or up to 50 specified times during that total period.

Standard methods can simply be called from disk and started within seconds. Help information, operator prompts, audio visual warnings and messages enable any operator, irrespective of skill level, to run the system and to obtain results with speed and confidence. Raw data,

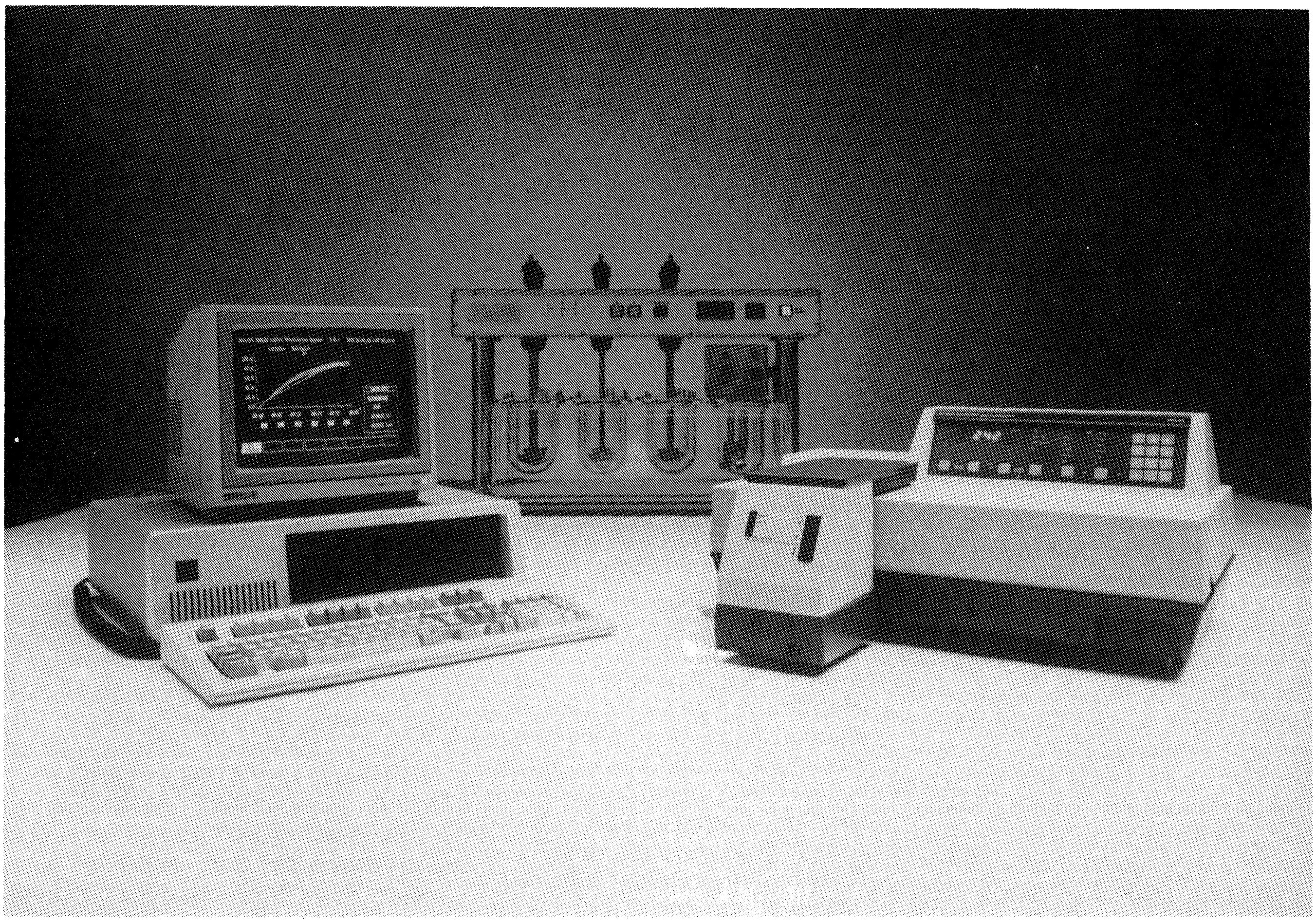

*Philips Analytical's new tablet dissolution system features multi-tasking software for maximum flexibility.*

best-fit curves, tables of results at set times and percentages are all readily available.

There are various method options to choose from, such as quality control, research, tablet weight normalization and time selection.

Data is stored automatically as the run progresses and can be displayed in real time or post run. Complete methods with all dissolution parameters, spectrophotometer settings and cell programmer controls can also be stored and recalled at any stage for altering, checking, setting or queuing.

The multi-tasking design of the PU8620 tablet dissolution system allows continuous access to all areas of the software, which means there is no need to waste the power of a personal computer on just collecting data over 24 hours.

The operator can view the last night's results or set up a new method whilst simultaneously running the dissolution monitoring process.

*Further information from Pye Unicam Ltd, York Street, Cambridge CB1 2PX, UK. Tel.: 0223 358866.*

### High flow peristaltic pumps

Watson-Marlow has produced a range of pumps which they consider energy efficient, versatile, powerful and extremely cost-effective. Within this 700 series are five distinct pump ranges, all of which share the same power-driven roller design 701R pumphead to provide flows up to 2000 l/h: the 701U/R has 50 : 1 speed control, is user-programmable to accept analogue voltage or current process control signals, and has a rugged die-cast casing; the 701S/R is identical to the 701U/R but does not have process-control facilities; the 701P/R is a variable speed pneumatic pump showing the same die-cast case; the 701FBC/R fixed-speed pumps and 701VB/R variable-speed pumps are base-plate mounted. Explosion-proof pumps are also available either in fixed or variable speed versions.

A 701RX extension pumphead may be fitted to any 700 series pump to double its maximum flow rate.

The 700 series is capable of pumping 2000 l/h using standard 25·4 mm bore tubing, and gives a tube life of at least five times that previously available. The use of Marprene tubing, which is food quality, extends tube life even further.

*Pump specifications including flow rates, are detailed in the Watson-Marlow catalogue – Copies from Smith and Nephew Watson-Marlow (see p. 144).*

### New from Du Pont

A number of products has recently been launched into the UK marketplace by Du Pont. The new introductions are listed below, following a short history of the company.

E. I. Du Pont de Nemours Co. Inc. is one of the world's largest industrial enterprises, employing 145 000 people in some 50 countries and sales of $30 billion. The company's interests include biomedical products and diagnostics, graphic arts and X-ray materials, electronics, paints and coatings, fibre technology, polymer products, agricultural and industrial chemicals, petroleum exploration, production, refining and marketing, and coal mining. Together these constitute over 1800 product lines; including such names as 'Nylon', 'Teflon', 'Lycra' and 'Mylar'.

The company is also one of the longest-established industrial concerns, with a history of over 180 years of operation. In 1802, a young French immigrant to the USA, Eleuthier Irenee du Pont de Nemours established a business on the twin principles of making 'products of undisputed worth' and that 'every employee should be treated with dignity and respect'. In 1804, he was granted a patent for granulating gunpowder, the first of nearly 50 000 patents since then. For the first century E I du Pont de Nemours & Co. was wholly or largely an explosives manufacturer, with a reputation for technical innovation and excellence. One of its innovations – the search for a smokeless powder – led to an interest in applications of nitrocellulose.

In 1903, Du Pont's first industrial research laboratory was established, and the company began to diversify. Nitrocellulose-based lacquers, coated fabrics and plastics were followed by acids, pigments and mixed paints. Next came synthetic dyes, cellulose films, rayon acetate fibre and ammonia.

In the early years of this century, Europe, particularly Germany and the UK, was pre-eminent in basic chemistry and production technology, and Du Pont's American operation specialized in improving existing products and processes. But in 1927, the company started fundamental research and made many important innovations now taken for granted, including the discovery of neoprene (the first multi-purpose synthetic rubber) and nylon (the original man-made fibre).

By its 150th year, Du Pont had expanded, by growth and acquisition, to making 1400 products. In 1981, it merged with Conoco, allowing diversification into energy and chemical feedstocks, oil, coal, natural gas, and the establishment of laboratories dedicated to research into life sciences, now a major growth area of the company's interests.

In 1986, Du Pont has over 1800 products, and employs 6000 research and development scientists and engineers in 80 laboratories worldwide, with an overall budget of over $1100 million – nearly double the 1979 R & D budget. The success of this commitment to research can be gauged by the 90 distinct new products introduced in the last 20 years as a result of discoveries in these labs.

Now Du Pont's long-term research goals are in the areas of life sciences, alternative energy sources, environmental and occupational health, new polymer and photo systems, more accurate instrumentation, innovations in processes, and fundamental research into molecular behaviour to underpin product and process improvements.

*Du Pont in the UK and Europe*

Du Pont's UK operation, established in 1956, was the first European subsidiary, and was rapidly followed by companies in all principal European countries. In the UK, Du Pont has its largest site at Maydown, Northern Ireland, now in its 26th year of operation and an elastomers research facility – also over 25 years old – in Hemel Hempstead. Both of these, and other Du Pont sites have won many national and international awards for occupational safety.

Products manufactured in Britain account for almost half of Du Pont UK's annual turnover, and exports are nearly £100 M. As part of Du Pont's policy of reinvestment, there is a new electronics component facility in Bristol, a planned electronics site in North Avon, and a high-efficiency coal-fired co-generation plant on construction in Maydown.

The majority of Du Pont's 50 companies in Europe are wholly-owned, some started from scratch, and some as a result of the Conoco merger or other acquisitions. They employ 18 000 people in 45 plants, over 20 laboratories, two refineries and in North Sea gas and oil exploration.

The investment in Europe is nearly £5 billion, the largest segment of Du Pont's non-USA investment. Part of this is due to the company's commitment to health and safety provision for its workforce, including measures to safeguard air and water purity.

*Interleuken-2 reagent for immune system studies*

Iodine-labelled rIL-2 ($^{125}$I rIL-2), a sensitive, recombinant form of Interleuken-2, is now offered for the analysis of IL-2 receptors on stimulated lymphocytes, which play a crucial role in activating the body's immune system. The inability of cells to produce or respond to IL-2 has been implicated in such diseases as autoimmunity, AIDS and cancer.

Du Pont's rIL-2 reagent is a cell growth factor that causes lymphocytes in the body's immune system to proliferate and activate so that they can fight foreign cells and proteins more efficiently. It is used to analyse the structure of both high- and low-affinity Il-2 receptors and to activate naturally occurring 'killer cells', which are currently being studied for their potential clinical applications.

Du Pont's $^{125}$I rIL-2 is also used in ligand-binding measurements to determine the proportion of high-affinity receptor sites, which relates directly to the body's ability to respond to IL-2.

Ligand-binding measurements also provide a convenient method for screening new synthetic compounds that may replace or modulate IL-2 for pharmacological purposes.

The iodinated $^{125}$I rIL-2 has a higher specific radioactivity than biosynthetically labelled IL-2 to further the study of high-affinity IL-2 binding sites.

*Monoclonal cytoskeletal staining kits*

The 'Cytolite' monoclonal cytoskeletal reagents kits can detect well-characterized actin, tubulin, vimentin and keratin antibodies. They all are specific against cytoskeletal proteins. All reagents are tested with normal and transformed cells, such as mouse mammary tumour and embryonic human skin.

Both a kit format or a separate reagent package are available which contain detailed, up-to-date procedures in rhodamine or fluorescein staining detection. Each provide sufficient antibody for 50 assays per vial.

*Large-scale peptide synthesis*

The 'Coupler' 296 is the largest capacity, solid-phase peptide synthesizer available. It consistently and economically produces up to 500 grams of peptide in a single run. For use in commercial-scale bio-research and for pharmaceutical applications, the 'Coupler' 296 performs a wide range of high-quality peptide syntheses within the rigorous requirements of good manufacturing practice. It uses common blocking groups such as BOC, benzyl, t-butyl and FMOC and coupling strategies such as DCC and active ester. The synthesizer is being used by major pharmaceutical manufacturers throughout the world for pilot and production-scale operations. Typical stepwise coupling efficiency exceeds 99% with a typical cleavage yield of 86.3%.

The system consists of a chemistry module, a driver module, a reaction module with two interchangeable 5 l reaction vessels for continuous operation and a microcomputer that automatically controls all instrument functions. The chemistry module offers random access to eight amino-acid reservoirs and eight solvent/reagent reservoirs.

*Coder 300*

Du Pont's Biotechnology Systems Divison introduced a computer automated system in September 1986 to provide rapid, efficient and reliable synthesis of oligonucleotides. The Coder 300 DNA synthesizer employs advanced B-cyanoethyldiosopropyl phosphoramidite chemistry and produces efficient reproducible syntheses of oligonucleotides on a scale of 0, 25, 1 or 5 micromoles. The Coder 300 controls fluid metering and microvalve plumbing to admit nucleotide derivations one by one in the order of an operator-dictated sequence. The growing DNA molecule is immobilized on a solid support. When the synthesis is complete, the DNA is recovered and purified for experimental use.

Simple software commands access preprogrammed procedures for reproducible results. This means that the operator does not need prior chemical training to synthesize DNA fragments successfully.

The Coder 300 is available in single and multi-column configurations. A fluid sensor monitors the delivery of all reagents in the system. A number of signal alerts are built into the system to help prevent loss of material or damage to the system. In the event of a power failure, an automatic restart resumes synthesis at the point the failure occurred when power is restored.

### BOOK REVIEW

### Chemical Pattern Recognition

By O. Strouf, Research Studies Press Ltd, Letchworth, UK (1986), (distributed by John Wiley and Sons Ltd). 202 + xvi pp. £26.65 ISBN 0 86380 044 0 (Wiley Inc. 0 471 91252 2).

Chemical pattern recognition is a major branch of chemometrics – a subject that has been receiving considerable popular attention recently. This book reviews progress in the subject over the years 1979 to 1985.

A brief introductory chapter provides a broad overview and prepares the ground for a chapter on methodology (comprising about 60% of the book) in which advances in pattern classification, feature selection and visualization techniques are surveyed. Of particular interest in the following chapter on applications are the sections devoted to the various branches of analytical spectroscopy and chromatography. A very brief final chapter reviews recent trends in a clear and informative way and gives one or two pointers to the future. The book concludes with a compilation of 436 references, the majority of which cover the years 1979 to 1985.

Although mathematical treatment is kept to a minimum, this book is not one that can be recommended to the beginner who is not reasonably well versed in the concepts and statistical techniques of chemometrics. It will, however, be a valuable survey for established chemometricians.

One grows, albeit reluctantly, used to books printed from camera-ready copy, and in a book such as this, which is addressed to a minority readership, the need to keep costs down must be an overriding factor. In such circumstances, therefore, more attention should be paid to the appearance of the page if strain on the reader is to be minimized. The author from the Czechoslovakian Academy of Sciences must be congratulated on his command of the English language. It is a pity that the British publisher and series editor appear to have given him only minimal assistance.

**Derrick G. Porter**

*Department of Trade and Industry,*
*Room 701B, 29 Bressenden Place,*
*London SW1 5DT*

Du Pont is also offering a line of DNA synthesis reagents consisting of cyano-ethylphosphoramide derivatives of the four nucleotide bases, controlled pore glass supports for synthesis and other solvents and reagents in bulk quantities.

### Portable nitrogen oxide analyser

A lightweight NOx analyser for measuring concentrations in flue emission and various combustion gases is now available in the UK.

To provide unparalleled stability and long service life, a semiconductor sensor in Shimadzu's NOA-305 is used in place of the conventional photomultiplier in the chemiluminescence detector.

Warm-up time is less than 5 min – conventional instruments which require two to three hours – and its full-scale measuring range is adjustable in five steps from 50 to 1000 ppm.

A full range of options is available with the machine, including sampling devices and various data displays.

*The UK distributor is Dyson Instruments Ltd, Hetton Lyons Industrial Estate, Hetton, Houghton-le-Spring, Tyne & Wear DH5 0RH, UK. Tel.: 0670 260452.*

### High-performance MS

The JMS-HX 110 is a high-performance mass spectrometer, which can be used in MS-MS tandem operation. The system is well-suited to new FAB ionization techniques for measurements on compounds with mass numbers between 3000 and 6000, including biological compounds such as glycolipids, proteins and peptides. In its MS-MS mode, besides providing accurate structure information on organic compounds, it can, unlike a single MS system, be used to determine an amino-acid sequence of polypeptide and for quantitative analysis of trace samples.

The ion optical geometry of the JMS-HX 110 is such that users can work on a wide mass range and obtain very high resolutions of over 100 000 without sacrificing sensitivity. Mass range of 12 500 daltons at maximum acceleration voltage of 10 kV and up to 125 000 daltons at 1 kV can be covered.

The system offers a broad range of ion sources including EI/CI; EI/CI/DCI; FAB/EI/CI/DCI and FAB/FD combinations. As well as the MS-MS interface, Jeol also provide interfaces for LC/MS and GC/MS. The system gives GC/MS sensitivity of 0.1 nanograms of methyl stearate in the EI mode and an ion intensity of $3 \times 10^{-8}$ coulombs/microgram with the molecular ion of methyl stearate.

The evacuation of the JMS-HX 110 is fully automatic, there being no need for manual valve operation.

Computer control of the system's operation is carried out by the Jeol DA 5000 data system. The data system corrects all parameters for runs in either positive or negative, controls temperature and acquires, tabulates and displays spectra in several ways including 3D chromatograms. It can highlight and compare selected parts of the spectra and slow scan over particular peaks of a single mass or two. It allows accurate mass calibration of the second magnetic sector enabling accurate daughter ion spectra to be obtained rapidly on-line over the full mass range.

*Further details are available from Jeol (UK) Ltd, Jeol House, Grove Park, Colindale, London NW9 0JN. Tel.: 01 205 6376.*

### HTLV-III assay

Du Pont has announced an HTLV-III Dipstick Assay for the detection of HTLV-III antibodies in serum or plasma samples. The easy-to-use screening test takes 45 min and indicates presence or absence of the HTLV-III antibody by an increase in colour intensity which can be detected visually or by measuring optical density with an absorbance meter.

The Dipstick test can be carried out without the need for investment in expensive laboratory equipment required for batch testing or mass screening. It is therefore particularly useful when small numbers of samples need to be tested. The Dipstick test contains all the necessary reagents for 50 assays, costing only £2 per test.

As with any screening test, a positive result obtained with the Dipstick Assay should be confirmed *independently*. Results obtained from the Dipstick Assay correlate well with those obtained from other HTLV ELISA kits. The incidence of false positives is low – approximately 0.5%.

To complement their existing range of HTVL-III confirmatory diagnostic tests Du Pont has also recently launched a Histocytochemical Kit for the detection of HTLV-III p24 core protein in infected cells or tissues.

*More information from Du Pont (UK) Ltd, Wedgwood Way, Stevenage, Hertfordshire SG1 4QN, UK. Tel.: 0438 734534.*

### Air sampling filters

There is increasing concern about the effects on human life of toxic particulates in the air. Sampling and analytical methods used to monitor air quality are becoming highly sophisticated. EPM 2000, a new Whatman filter, was designed and developed to complement these methods as a medium for advanced air filtration, suitable both for quantitative and qualitative measurements.

This filter was developed for use with Hi-vol air samplers and possesses such advanced technical characteristics as high chemical purity, high air flow rate and excellent particle retention efficiency. These technical characteristics combine to set a new standard for Hi-vol air sampling filter media.

The EPM 2000 is manufactured from 100% borosilicate glass microfibre, under strictly controlled conditions. It is heat-treated after manufacture to remove organic traces and no binder is used to give maximum purity. Individually numbered sheets for rapid identification and recording.

*Details from Whatman Ltd, Springfield Mill, Maidstone, Kent ME14 2LE, UK. Tel.: 0622 61681.*

